# Mental health of primary health care physicians and nurses following prolonged infection control rules: a national survey in China

**DOI:** 10.3389/fpubh.2024.1392845

**Published:** 2024-08-23

**Authors:** Crystal Jingru Li, Yanling Zheng, Yong Gan, Zhaohui Du, Xuemin Cai, Yongjin Li, Wei Wang, Tianwu Jiang, Qingyu Zhang, Lei Niu, Tiffany Junchen Tao, Wai Kai Hou

**Affiliations:** ^1^Centre for Psychosocial Health, The Education University of Hong Kong, Hong Kong, Hong Kong SAR, China; ^2^Shouyilu Street Community Health Service Center, Wuhan, Hubei, China; ^3^Department of Social Medicine and Health Management, School of Public Health, Tongji Medical College, Huazhong University of Science and Technology, Wuhan, Hubei, China; ^4^Department of Administrative Management, Shanggang Community Health Service Center, Shanghai, China; ^5^Department of Administrative Management, Laoshan Community Health Service Center, Qingdao, Shandong, China; ^6^Department of Administrative Management, Jinsong Community Health Service Center, Beijing, China; ^7^Department of Administrative Management, Xinhua Shaocheng Community Health Service Center, Chengdu, Sichuan, China; ^8^Department of Administrative Management, Tianshui Wulin Street Community Health Service Center, Hangzhou, Zhejiang, China; ^9^Department of Administrative Management, Jiexin Village Community Health Service Center, Lanzhou, Gansu, China; ^10^Department of Administrative Management, Xinglin Street Community Health Service Center, Hefei, Anhui, China; ^11^Department of Psychology, The Education University of Hong Kong, Hong Kong, Hong Kong SAR, China

**Keywords:** COVID-19, mental health disorder, primary healthcare physician and nurse, post-pandemic, SCL-90-R, post-traumatic stress disorder, insomnia

## Abstract

**Introduction:**

This study examined the prevalence and correlates of probable mental health disorders, including psychological distress, somatization, depression, anxiety, phobic anxiety (PHO), obsessive-compulsive disorder (OCD), post-traumatic stress disorder (PTSD), and insomnia among Chinese primary health care (PHC) physicians and nurses amid the post-pandemic period in 2022.

**Method:**

Region-stratified sampling was conducted to recruit a national sample of 4,246 respondents from 31 July 2022 to 12 August 2022. A total of 692 primary healthcare institutions were identified in 30 provincial-level administrative regions of China. An online questionnaire was used for assessing probable mental health disorders using Symptoms Checklist-90-Revised (SCL-90-R) and PTSD Checklist for DSM-5 (PCL-5), and sleeping problems using Insomnia Severity Index (ISI). Data on demographics and work were also collected. Bivariate analysis and multiple logistic regression were conducted to identify significant correlates of probable mental health disorders.

**Results:**

A total of 4,246 valid questionnaires were identified. Results showed that relative to the prevalence of probable mental health disorders among health care workers at the early stage of the pandemic in China, there was an overall decreased prevalence except for somatization, PHO, and OCD among the current PHC physicians and nurses. Multiple logistic regressions showed that significant risk factors of common probable mental health disorders, namely psychological distress, SOM, DEP, ANX, PHO, OCD, PTSD, and insomnia, were female gender, multimorbidity, history of psychiatric disorders, quarantine experience, never asking anyone for help, and overtime work.

**Conclusion:**

Attention should be given to preexisting psychiatric and multimorbid conditions, social support, and work-related stressors. Regular assessment and psychological interventions are needed to enhance the mental health of PHC professionals even after public health crisis.

## 1 Introduction

Starting from the lockdown of Wuhan on January 23rd, 2020, China adopted some of the most prolonged and stringent infection control policies compared with other countries, including widespread regional lockdown, large-scale quarantine, mass temperature screening, community closed management, and extensive public health education in the community (1). China further implemented a dynamic zero-COVID policy to minimize COVID-19 transmission starting August 2021 until the abrupt lifting of all infection control rules on 26th December, 2022 (2). The outbreak of the COVID-19 pandemic has brought a substantial and prolonged crisis across the globe, characterized by high infection risk and death rates and could be defined as a traumatic event to all affected people especially those who have either been infected or have suffered from serious medical conditions due to the pandemic, including primary health care (PHC) workers (3–5). The prolonged infection control created significant challenges for Chinese PHC physicians and nurses. Different from the medical professionals at secondary and tertiary hospitals in China, PHC physicians and nurses were the important cornerstone of the healthcare system and the first point of contact for individuals and families in the community, who have taken up the key role of implementing the infection control rules such as diagnosis, triage, and monitoring in collaboration with hospitals and the health department (6–8). Prolonged infection control has been found to create extra challenges to PHC physicians and nurses (6, 9), including overtime work (working hours ≥50 h per week) (10), night duty number ≥10 (11), long shift workhours (8–12 h) (12), working in high-risk environment (e.g., working front-line, working in hardest hit area, or providing direct care to infected patients) (6), and providing direct care to infected patients in ICU or Fangcang shelter hospitals (13, 14).

During the pandemic, frontline healthcare workers could experience continued physical and psychological distress due to prolonged control rules and challenges in multiple stressful and high-risk work environments (6, 11, 15–22). Initial evidence has been gathered upon high probable mental health disorders among Chinese PHC physicians and nurses (5, 23, 24), consistent with the high prevalence of probable mental health disorders across different stages of the pandemic among Chinese medical workers (4). It remains unclear whether the high prevalence was consistent across regions, urban and rural settings, and demographic and occupational characteristics (8, 25). Such evidence will be of global relevance for optimizing the resilience of the primary care system for any future pandemic or large-scale disasters (26, 27).

Previous studies in other regions have identified sociodemographic factors of mental health disorders including younger age, female gender, being unmarried, having children or dependents to care, preexisting chronic disease(s), and histories of psychiatric disorders (13, 15, 20, 21, 27, 30, 31). Apart from demographics, risk factors could be significant risk factors of mental health disorders. Among healthcare professionals in Canada, other health care workers relative to physicians have been found to report higher levels of symptoms of depression, anxiety, and perceived (28). Specifically, nurses have been consistently found to be more vulnerable to psychological problems than physicians across regions (6, 14, 23, 29). Technical and administrative staff have been demonstrated to experience higher odds of common mental disorders, including depression, anxiety disorders, post-traumatic stress disorder, panic attacks, and substance use disorder (13). In addition, redeployment, lack of specialized training, and insufficient relevant medical work experience were found to be associated with higher symptoms of anxiety, burnout, depression, and PTSD (15, 18, 21).

The current study aims to investigate the prevalence of probable mental health disorders, including, somatization, depression, anxiety, phobic anxiety, obsessive-compulsive disorder, PTSD, and insomnia and their sociodemographic and occupational correlates among a national sample of PHC physicians and nurses amid the post-pandemic era in China. This study hypothesizes that probable mental health disorders are more common among PHC physicians and nurses in regions with a more significant impact of the pandemic and concomitant infection control (15, 32) and the prevalence of probable mental disorders are positively associated with sociodemographic and work-related risk factors.

## 2 Materials and methods

### 2.1 Study design and respondents

**Figure 1:**
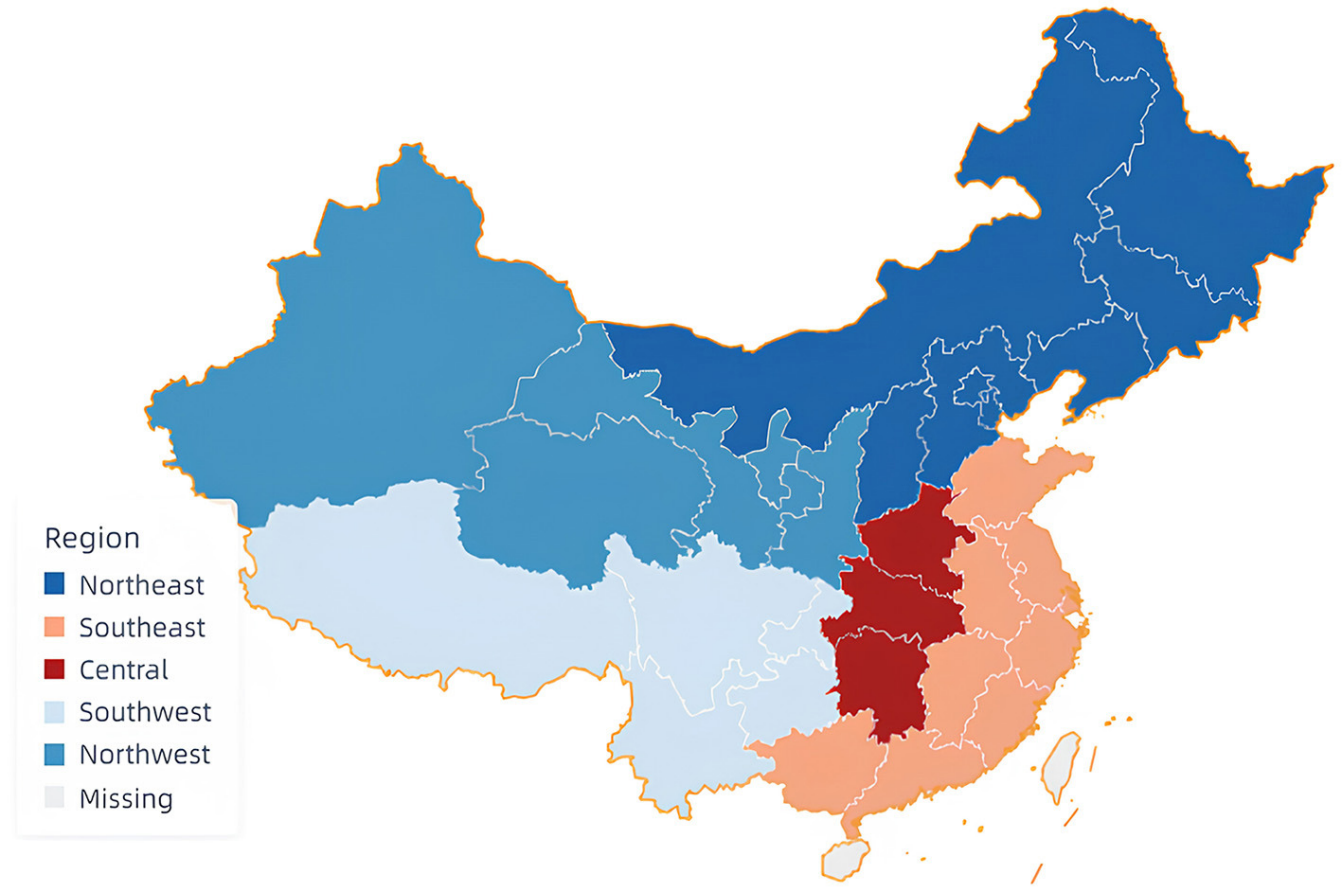
The five regions of China participated in the current study.

Upon obtaining the ethics committee's approval from The 7th Hospital of Wuhan (220701), respondents were recruited using region-stratified sampling. The sampling regions are shown in Figure 1. Five geographical regions of China were covered, including the central (3 provinces), southeast (7 provinces, 1 autonomous region, 1 municipality), southwest (3 provinces, 1 autonomous region, 1 municipality), northeast (5 provinces, 1 autonomous region, 2 municipalities), and northwest (3 provinces, 2 autonomous regions) (33). A total of 30 administrative regions (21 provinces, 5 autonomous regions, 4 municipalities) were covered.

Recruitment of respondents and administration of the survey was conducted online via Wenjuanxing, a widely used platform in China, from July 31-August 12, 2022. Electronic questionnaires were delivered through the Community Health Center Directors' Alliance affiliated with the Community Health Association of China, which is a national first-level organization under the management of the Ministry of Civil Affairs and the National Health Commission. Inclusion criteria were (1) age 18 years or above and (2) PHC physicians and nurses. Respondents were randomly selected using a disproportionate stratified sampling design with nine strata defined according to gender (male and female), occupation (physician and nurse), and technician title (none, junior, intermediate, associate senior, and senior). To ensure that the current sample was representative of PHC physicians and nurses in China (34), the current study (1) oversampled female respondents, (2) recruited physicians and nurses in the ratio of approximately 1:1, and (3) recruited respondents with junior and intermediate technician title based on the proportion of healthcare professionals as outlined in previous PHC national sample in China (35). Electronic informed consent was obtained from all respondents before the survey. All questions in the questionnaire needed to be answered before submission resulting in the absence of missing data. A returned survey was considered invalid if (1) no electronic informed consent, (2) the average response time on each item was < 2 s (34), or (3) maximum long string (i.e., consecutive identical responses) was equal to or larger than half of the item number (35, 36).

### 2.2 Measurements

#### 2.2.1 Background variables

Demographic data were collected, including gender, age, province, residence (rural or urban), workplace (i.e., community health center or station, village and township clinics), education level (i.e., below bachelor, bachelor, and master or above), marital status (i.e., unmarried and married), childcare responsibility (yes or no, age of the child is required to be indicated), and any diagnosed chronic medical conditions (i.e., no, hypertension, diabetes, chronic obstructive pulmonary disease, pneumonia, chronic bronchitis, stroke, and others). They also reported their occupation (i.e., doctor, nurse), technical title (i.e., none, junior, intermediate, associate senior, and senior), and frequency of overtime/longer than 8 hours of work (not at all, sometimes, more than half the time, and almost every day). Respondents also rated fear of infecting COVID-19 on duties (strongly disagree, disagree, neutral, agree, strongly agree) and the cumulative number of quarantines (0, 1, 2, 3, or more than 3).

#### 2.2.2 Probable mental health disorders

Probable mental health disorders were measured using the Chinese version of Symptoms Checklist-90-Revised (SCL-90-R) (37), the 20-item PTSD Checklist for DSM-5 (PCL-5) (38), and the 7-item Insomnia Severity Index (ISI) (39).

##### 2.2.2.1 SCL-90-R

Respondents rated on a 5-point scale (0 = not at all, 1 = a little bit, 2 = moderately, 3 = quite a bit, 4 = extremely) for nine dimensions of psychological distress, including somatization (SOM), obsessive-compulsive disorder (OCD), interpersonal sensitivity, depression (DEP), anxiety (ANX), hostility, phobic anxiety (PHO), paranoid ideation, psychoticism, and seven additional items that assess appetite and sleep disturbances. The scores of all 90 items were averaged to form the Global Severity Index (GSI) to indicate overall psychological distress. GSI scores ≥1 indicated probable general psychological distress (37). Average scores were also calculated for SOM, DEP, ANX, PHO, and OCD. Raw average scores were then converted into T-scores (mea*n* = 50, SD = 10) based on the mean scores of prior representative samples of Chinese medical workers (40). Subscale T-scores ≥63 were identified as high psychological distress/probable mental disorders (subscales) (41). SCL-90-R has been found to be valid and reliable for assessing mental health symptoms in Chinese populations (42). In the current study, Cronbach's α of the SCL-90-R = 0.990. The Cronbach's α for the selected subscales showed good internal consistency: SOM = 0.929, DEP = 0.942, ANX = 0.935, PHO = 0.898, and OCD = 0.922.

##### 2.2.2.2 PCL-5

Respondents reported their experiences and responses to the threat of traumatic events in the past month on a 5-point scale (0 = not at all, 4 = extremely). A cutoff score of 33 or above was used to indicate probable PTSD (43). PCL-5 has been shown to have excellent internal consistency, test-retest reliability, and convergent and discriminant validity (44). The valid and reliable Chinese version of PCL-5 was widely used across Chinese populations (45). The Cronbach's α was 0.978 in the current administration.

##### 2.2.2.3 ISI

The index assessed the nature, severity, and impact of insomnia on a 5-point scale (0 = none, 1 = mild, 2 = moderate, 3 = severe, and 4 = very severe). A cutoff score of 10 was used to indicate probable insomnia (39). ISI is a valid and reliable instrument for detecting insomnia among Chinese health care workers (6). In the present study, the Cronbach's α = 0.822.

#### 2.2.3 Workplace social support

Social support in the workplace was assessed using selected items from the Social Support Rating Scale (46), including emotional support from colleagues (You and your colleagues: 1 = Never care for each other, just nod to each other, 4 = Most colleagues care about you), seeking emotional support (The way you confide when you have trouble: 1 = Never tell anyone, 4 = Confide your troubles voluntarily to get support and understanding), and seeking instrumental support (The way you seek help when you are in trouble: 1 = Only rely on themselves and do not accept help from others, 4 = Frequently ask for help from family, relatives, and organizations when in trouble). Higher scores indicated higher levels of social support and support-seeking. Social Support Rating Scale has shown good validity and reliability across Chinese populations (26, 47). In the present study, the alpha was 0.788.

### 2.3 Statistical analysis

First, we examined the prevalence of different probable mental health disorders. Nonparametric Mann-Whitney U tests were conducted to investigate sociodemographic and occupational correlates of mental health disorders. Next, multiple logistic regressions were conducted to test the correlates that were significant in previous bivariate analyses within a multivariable environment. Adjusted odds ratios (aORs) with 95% CI were reported. SPSS 26.0 (RRID:SCR_002865) was used for all analyses.

## 3 Results

### 3.1 Respondents and descriptive characteristics

The sampling frame consisted of 692 primary healthcare institutions, with 518 in urban areas and 174 in rural areas. A total of 5,826 online surveys were received, among which 4,246 (72.9%) were identified as valid data. Demographics did not differ between respondents with and without a valid survey (gender: *p* = 0.565, age: *p* = 0.046, marital status: *p* = 0.006, education level: *p* = 0.423, region: *p* = 0.037, residence: *p* = 0.785). The sample (37.8 ± 9.2 years of age) consisted of 2,120 physicians (49.9%) and 2,126 nurses (50.1%), among which 3,429 (80.8%) were female. The gender ratio was consistent with the ratio of 1:3 among Chinese primary medical workers (48). A total of 2,095 (49.3%) were in general practice; 3,575 (84.2%) worked in community health service centers and the remainder (*n* = 671, 15.8%) in village and township clinics. The majority of the respondents had never been quarantined (*n* = 3,113, 73.3%) and did not indicate fear of COVID-19 (*n* = 1,133, 88.9%). More than 60% reported overtime work sometimes (*n* = 2,744, 64.6%) and about 10% reported overtime work almost every day (*n* = 420). Demographics and study variables are summarized in Table 1.

**Table 1:** Sociodemographic characteristics of PHC physicians and nurses.

**Variables**	***n*** **(%)**	
**Sex**		
Male	817	(19.2)
Female	3,429	(80.8)
**Age [Mean (SD)]**	37.84	(9.24)
18–34	1,715	(28.8)
35–49	1,960	(41.2)
50 or above	571	(53.3)
**Marital status**		
Unmarried	787	(18.5)
Married	3,459	(81.5)
**Living region**		
Central	1,462	(34.4)
Southeast	1,427	(33.6)
Southwest	646	(15.2)
Northeast	327	(7.7)
Northwest	384	(9.0)
**Living areas**		
Urban	3,565	(84.0)
Rural	681	(16.0)
**Education**		
Below bachelor	1,545	(36.4)
Bachelor or above	2,701	(63.6)
**Workplace**		
Village and township clinics	671	(15.8)
Community health service center	3,575	(84.2)
**Occupation**		
Doctor	2,120	(49.9)
Nurse	2,126	(50.1)
**Doctor job position**		
Not applicable	2,126	(50.1)
Front-line	2,033	(47.9)
Non-front-line	87	(2.0)
**Technical title**		
None	364	(8.6)
Junior	1,731	(40.8)
Intermediate	1,652	(38.9)
Associated senior	442	(10.4)
Senior	57	(1.3)
**Overtime work**		
Never	278	(6.5)
Sometimes	2,744	(64.6)
More than half the time	804	(18.9)
Almost everyday	420	(9.9)
**Childcare responsibility**		
No	1,774	(41.8)
Yes	2,472	(58.2)
**Chronic disease**		
No	3,301	(77.7)
Yes	945	(22.3)
**Multimorbidity**		
No	3,927	(92.5)
Yes	319	(7.5)
**Psychiatric history**		
No	4,161	(98)
Yes	85	(2.0)
**Afraid of COVID-19**		
No	3,772	(88.8)
Yes	474	(11.2)
**Cumulative quarantine times**		
0	3,113	(73.3)
1	641	(15.1)
2	259	(6.1)
≥3	233	(5.5)
**Emotional support from colleague**		
Care to some extent	4,028	(94.9)
Never care about each other	218	(5.1)
**Emotional support-seeking behavior**		
Tell others	3,783	(88.1)
Never tell anyone	463	(10.9)
**Instrumental support-seeking behavior**		
Ask others for help	3,794	(89.4)
Never ask anyone for help	452	(10.6)

SD, standard deviation; PHC, primary health care.

### 3.2 Prevalence of mental health disorders

As shown in Table 2, among the 4,246 respondents, 11.5% of the respondents reported general psychological distress (95%CI = 10.6, 12.5), 11.2% (95%CI = 10.3, 12.2) reported somatization, 8.2% (95%CI = 7.4, 9.1) reported probable depression, 5.9% (95%CI = 5.2, 6.6) reported probable anxiety, 6.2% (95%CI = 5.5, 7.0) reported probable phobic anxiety, 7.9% (95%CI = 7.1, 8.8) reported probable OCD, 6.8% (95%CI = 6.0, 7.6) reported probable interpersonal sensitivity, 9.0% (95%CI = 8.2, 8.9) reported probable hostility, 6.2% (95%CI = 5.5, 6.9) reported probable paranoid ideation, 7.0% (95%CI = 6.2, 7.7) reported probable psychoticism, 6.9% (95%CI = 6.1, 7.7) reported probable PTSD, and 18% (95%CI = 16.9, 19.2) reported probable insomnia.

**Table 2:** Prevalence of current probable mental health disorders among primary health care physicians and nurses.

	**PHC physicians and nurses (*****n*** = **4,246)**	
	***n*** **(%)**	
Psychological distress	489	(11.5)
Somatization	476	(11.2)
Depression	349	(8.2)
Anxiety	250	(5.9)
Phobic anxiety	263	(6.2)
Obsessive-compulsive disorder	336	(7.9)
Interpersonal sensitivity	288	(6.8)
Hostility	384	(9.0)
Paranoid ideation	262	(6.2)
Psychoticism	297	(7.0)
Post-traumatic stress disorder	292	(6.9)
Insomnia	766	(18.0)
Absence	2,903	(68.4)
Sub-threshold	1,118	(26.3)
Moderate	183	(4.3)
Severe	42	(1.0)

Psychological distress was defined when GSI score ≥1 in SCL-90-R; probable mental health disorders were defined when subscale T-scores ≥63 in SCL-90-R.

### 3.3 Correlates of probable mental health disorders

The multiple results of the logistic regression analyses are presented in Table 3. Female gender, multimorbidity, psychiatric histories, quarantine experience, working overtime for half of the time or more, and never asking help were associated with increased odds of the following outcomes: probable general psychological distress (aOR = 1.54–7.06, 95%CI = 1.14–4.41, 2.08–11.31), somatization (aOR = 1.86–5.52, 95%CI = 1.37–4.41, 2.52–8.89), depression (aOR = 1.59–8.97, 95%CI = 1.25–5.55, 2.56–14.49), anxiety (aOR = 1.47–9.73, 95%CI = 1.10–5.88, 1.96–16.07), phobic anxiety (aOR = 1.58–6.84, 95%CI = 1.20–4.12, 2.07–11.37), OCD (aOR = 1.50–7.13, 95%CI = 1.17–4.40, 1.92–11.55), PTSD (aOR = 1.48–6.97, 95%CI = 0.99–4.25, 2.21–11.44), and insomnia (aOR = 1.38–5.96, 95%CI = 1.08–3.73, 1.76–9.54). In addition, older age was associated with increased odds of probable somatization (aOR = 1.02, 95%CI = 1.00, 1.03), whereas having a bachelor's degree or above was associated with increased odds of probable general psychological distress (aOR = 1.43, 95%CI = 1.11, 1.82), somatization (aOR = 1.39, 95%CI = 1.08, 1.78), OCD (aOR = 1.39, 95%CI = 1.04, 1.86), interpersonal sensitivity (aOR = 1.25, 95%CI = 1.02, 1.52), psychoticism (aOR = 1.45, 95%CI = 1.12, 1.88), and insomnia (aOR = 1.29, 95%CI = 1.06, 1.57). Relative to respondents living in the Central region, those living and working in the Southeast region were more likely to report probable somatization (aOR = 1.39, 95%CI = 1.08, 1.79), and PTSD (aOR = 1.49, 95%CI = 1.09, 2.04). Those living in the Southwest region were more likely to report probable general psychological distress (aOR = 1.48, 95%CI = 1.10, 2.01), somatization (aOR = 1.52, 95%CI = 1.12, 2.07), hostility (aOR = 1.31, 95%CI = 1.01, 1.70), paranoid ideation, (aOR = 1.43, 95%CI = 1.07, 1.92), psychoticism (aOR = 1.68, 95%CI = 1.21, 2.33), and PTSD (aOR = 1.51, 95%CI = 1.03, 2.21). Respondents with colleagues never caring about each other at work reported increased odds of probable phobic anxiety (aOR = 2.00, 95%CI = 1.30, 3.07), interpersonal sensitivity (aOR = 1.48, 95%CI = 1.05, 2.09), psychoticism (aOR = 1.65, 95%CI = 1.12, 2.42), and PTSD (aOR = 1.72, 95%CI = 1.11, 2.66) relative to respondents with colleagues caring to some extent. Finally, those never confiding their troubles to others reported increased odds of psychological distress (aOR = 1.85, 95%CI = 1.34, 2.56), probable somatization (aOR = 1.73, 95%CI = 1.24, 2.42), depression (aOR = 1.79, 95%CI = 1.24, 2.58), anxiety (aOR = 2.07, 95%CI = 1.37, 3.12), phobic anxiety (aOR = 1.75, 95%CI = 1.17, 2.62), and OCD (aOR = 1.91, 95%CI = 1.32, 2.77). Results from bivariate analyses are summarized in Supplementary material 1.

**Table 3:** Multiple logistic regression of risk factors associated with probable mental health disorders and insomnia.

**Variable**	**Adjusted odds ratio (95% confidence interval)**					
	**Psychological distress**	**Probable somatization**	**Probable depression**	**Probable anxiety**	**Probable phobic anxiety**	**Probable obsessive-compulsive disorder**
**Gender**						
Male	Ref	Ref	Ref	Ref	Ref	Ref
Female	**1.54 (1.14–2.08)**	**1.86 (1.37–2.52)**	**1.82 (1.29–2.56)**	**1.75 (1.17–2.61)**	**1.73 (1.16–2.59)**	**1.69 (1.19–2.40)**
**Age**	1.00 (0.99–1.02)	**1.02 (1.00–1.03)**	0.99 (0.98–1.01)	1.00 (0.98–1.02)	1.00 (0.98–1.02)	1.00 (0.98–1.02)
**Region**						
Central	Ref	Ref	Ref	Ref	Ref	Ref
Southeast	1.28 (0.99–1.64)	**1.39 (1.08–1.79)**	1.29 (0.96–1.72)	1.12 (0.79–1.60)	1.22 (0.87–1.71)	1.19 (0.89–1.6)
Southwest	**1.48 (1.10–2.01)**	**1.52 (1.12–2.07)**	1.24 (0.86–1.78)	1.48 (0.98–2.21)	1.36 (0.91–2.04)	1.36 (0.95–1.94)
Northeast	1.34 (0.90–1.98)	1.43 (0.96–2.12)	1.36 (0.86–2.14)	1.38 (0.82–2.34)	1.62 (1.00–2.64)	1.32 (0.83–2.08)
Northwest	1.17 (0.81–1.71)	1.27 (0.87–1.85)	1.29 (0.85–1.97)	1.29 (0.79–2.10)	1.43 (0.90–2.26)	1.23 (0.80–1.88)
**Residence**						
Rural	Ref	Ref	Ref	Ref	Ref	Ref
Urban	0.85 (0.63–1.15)	0.85 (0.62–1.15)	0.94 (0.67–1.33)	0.89 (0.59–1.34)	0.88 (0.60–1.31)	0.79 (0.55–1.13)
**Education level**						
Below bachelor	Ref	Ref	Ref	Ref	Ref	Ref
Bachelor or above	**1.43 (1.11–1.82)**	**1.39 (1.08–1.78)**	1.19 (0.89–1.59)	1.30 (0.92–1.83)	1.30 (0.95–1.81)	**1.39 (1.03–1.86)**
**Occupation**						
Doctor	Ref	Ref	Ref	Ref	Ref	Ref
Nurse	1.15 (0.90–1.47)	1.02 (0.80–1.30)	0.84 (0.64–1.12)	0.89 (0.64–1.25)	1.06 (0.77–1.45)	0.96 (0.73–1.28)
**Overtime work**						
Never and sometimes	Ref	Ref	Ref	Ref	Ref	Ref
More than half the time and almost everyday	**2.28 (1.85–2.81)**	**2.59 (2.10–3.19)**	**2.38 (1.87–3.04)**	**2.80 (2.11–3.71)**	**2.10 (1.60–2.76)**	**2.42 (1.90–3.08)**
**Chronic disease**						
No	Ref	Ref	Ref	Ref	Ref	Ref
Yes	1.02 (0.80–1.30)	0.94 (0.73–1.20)	0.97 (0.73–1.29)	0.87 (0.62–1.22)	0.97 (0.70–1.33)	0.94 (0.70–1.25)
**Multimorbidity**						
No	Ref	Ref	Ref	Ref	Ref	Ref
Yes	**1.85 (1.33–2.58)**	**2.32 (1.69–3.18)**	**1.94 (1.34–2.82)**	**2.38 (1.57–3.61)**	**1.81 (1.18–2.77)**	**2.09 (1.44–3.02)**
**Psychiatric history**						
No	Ref	Ref	Ref	Ref	Ref	Ref
Yes	**7.06 (4.41–11.31)**	**5.52 (3.42–8.89)**	**8.97 (5.55–14.49)**	**9.73 (5.88–16.07)**	**6.84 (4.12–11.37)**	**7.13 (4.40–11.55)**
**Quarantine experience**						
No	Ref	Ref	Ref	Ref	Ref	Ref
Yes	**1.64 (1.33–2.03)**	**1.68 (1.36–2.08)**	**1.59 (1.25–2.03)**	**1.47 (1.10–1.96)**	**1.58 (1.20–2.07)**	**1.50 (1.17–1.92)**
**Care from colleagues**						
Care to some extent	Ref	Ref	Ref	Ref	Ref	Ref
Never care about each other	1.40 (0.96–2.05)	1.25 (0.84–1.85)	1.48 (0.97–2.25)	1.49 (0.93–2.36)	**2.00 (1.3–3.07)**	1.34 (0.87–2.06)
**Emotional support-seeking behavior**						
Tell others	Ref	Ref	Ref	Ref	Ref	Ref
Never tell anyone	**1.85 (1.34–2.56)**	**1.73 (1.24–2.42)**	**1.79 (1.24–2.58)**	**2.07 (1.37–3.12)**	**1.75 (1.17–2.62)**	**1.91 (1.32–2.77)**
**Instrumental support-seeking behavior**						
Ask others for help	Ref	Ref	Ref	Ref	Ref	Ref
Never ask anyone for help	**2.10 (1.53–2.88)**	**1.94 (1.40–2.68)**	**2.20 (1.53–3.14)**	**2.35 (1.57–3.53)**	**2.33 (1.57–3.44)**	**1.88 (1.30–2.71)**
**Gender**						
Male	Ref	Ref	Ref	Ref	Ref	Ref
Female	1.42 (1.10,1.83)	1.48 (1.15,1.92)	1.34 (1.01,1.79)	1.19 (0.87,1.632)	**1.98 (1.35-2.90)**	**1.38 (1.08-1.76)**
Age	1.00 (0.99, 1.01)	1.01 (1.00, 1.02)	1.01 (0.99, 1.02)	1.00 (0.99, 1.02)	1.00 (0.98–1.02)	1.01 (0.99–1.02)
**Region**						
Central	Ref	Ref	Ref	Ref	Ref	Ref
Southeast	1.19 (0.96, 1.47)	1.22 (0.99, 1.51)	1.10 (0.86, 1.40)	1.31 (0.99, 1.72)	**1.49 (1.09–2.04)**	1.20 (0.98–1.47)
Southwest	1.27 (0.98, 1.66)	**1.31 (1.01, 1.70)**	**1.43 (1.07, 1.92)**	**1.68 (1.21, 2.33)**	**1.51 (1.03–2.21)**	1.23 (0.95–1.57)
Northeast	1.13 (0.80, 1.60)	**1.55 (1.12, 2.13)**	1.39 (0.96, 2.01)	**1.61 (1.06, 2.42)**	1.25 (0.75–2.07)	1.18 (0.86–1.64)
Northwest	0.97 (0.70, 1.36)	0.99 (0.71, 1.37)	1.06 (0.73, 1.52)	1.31 (0.88, 1.96)	1.16 (0.72–1.87)	1.02 (0.75–1.39)
**Residence**						
Rural	Ref	Ref	Ref	Ref	Ref	Ref
Urban	0.94 (0.73, 1.21)	0.98 (0.76, 1.25)	0.89 (0.67, 1.19)	0.95 (0.69, 1.30)	1.05 (0.73–1.51)	0.93 (0.73–1.18)
**Education level**						
Below bachelor	Ref	Ref	Ref	Ref	Ref	Ref
Bachelor or above	**1.25 (1.02, 1.52)**	1.41 (1.15, 1.72)	1.53 (1.22, 1.93)	**1.45 (1.12, 1.88)**	1.20 (0.88–1.63)	**1.29 (1.06–1.57)**
**Occupation**						
Doctor	Ref	Ref	Ref	Ref	Ref	Ref
Nurse	1.03 (0.84, 1.27)	1.14 (0.93, 1.40)	1.04 (0.83, 1.32)	1.10 (0.84, 1.43)	0.89 (0.66–1.19)	1.06 (0.87–1.29)
**Overtime work**						
Never and sometimes	Ref	Ref	Ref	Ref	Ref	Ref
More than half the time and almost everyday	1.86 (1.55, 2.24)	2.22 (1.85, 2.65)	2.00 (1.63, 2.46)	2.10 (1.67, 2.63)	**2.35 (1.81–3.05)**	**2.30 (1.93–2.73)**
**Chronic disease**						
No	Ref	Ref	Ref	Ref	Ref	Ref
Yes	1.04 (0.85, 1.29)	1.06 (0.86, 1.30)	1.01 (0.80, 1.28)	1.14 (0.88, 1.48)	1.18 (0.88–1.59)	0.85 (0.69–1.05)
**Multimorbidity**						
No	Ref	Ref	Ref	Ref	Ref	Ref
Yes	**1.54 (1.14, 2.08)**	**1.45 (1.08, 1.96)**	**1.67 (1.20, 2.31)**	**1.73 (1.22, 2.46)**	**1.66 (1.12–2.48)**	**1.63 (1.22–2.17)**
**Psychiatric history**						
No	Ref	Ref	Ref	Ref	Ref	Ref
Yes	8.07 (5.03, 12.96)	**6.96 (4.33, 11.18)**	5.27 (3.31, 8.41)	7.45 (4.63, 11.97)	**6.97 (4.25–11.44)**	**5.96 (3.73–9.54)**
**Quarantine experience**						
No	Ref	Ref	Ref	Ref	Ref	Ref
Yes	1.38 (1.15, 1.67)	1.33 (1.10, 1.6)	1.58 (1.28, 1.94)	**1.44 (1.14, 1.81)**	**1.65 (1.27–2.15)**	**1.56 (1.31–1.86)**
**Care from colleagues**						
Care to some extent	Ref	Ref	Ref	Ref	Ref	Ref
Never care about each other	**1.48 (1.05, 2.09)**	1.29 (0.91, 1.83)	1.90 (1.33, 2.70)	**1.65 (1.12, 2.42)**	**1.72 (1.11–2.66)**	1.35 (0.96–1.90)
**Emotional support-seeking behavior**						
Tell others	Ref	Ref	Ref	Ref	Ref	Ref
Never tell anyone	1.73 (1.29, 2.31)	**1.45 (1.08, 1.95)**	**1.62 (1.18, 2.23)**	**1.43 (1.01, 2.02)**	1.48 (0.99–2.21)	**1.50 (1.13–1.99)**
**Instrumental support-seeking behavior**						
Ask others for help	Ref	Ref	Ref	Ref	Ref	Ref
Never ask anyone for help	**1.59 (1.19, 2.13)**	1.76 (1.32, 2.35)	1.92 (1.41, 2.63)	2.64 (1.90, 3.67)	**2.39 (1.63–3.50)**	**1.41 (1.06–1.87)**

Boldface indicates statistical significance (p < 0.05), Ref refers to reference group.

## 4 Discussion

This study aims to investigate the prevalence of probable mental health disorders, and identify sociodemographic and work-related risk factors of the prevalence in a national sample of primary health care (PHC) physicians and nurses after a prolonged period of stringent infection control rules in China. The prevalence of probable mental health disorders ranged from 5.9% to 18%. Female gender, multimorbidity, psychiatric histories, and quarantine experience were related to increased odds of common probable mental health disorders, namely SOM, DEP, ANX, PHO, OCD, PTSD, and insomnia. On top of these sociodemographics were work-related factors including frequent working overtime, and never asking for help at work.

### 4.1 Prevalence of mental health disorders

Two years after the COVID-19 pandemic outbreak, the prevalence of probable depression (8.2%), anxiety (5.9%), PTSD (6.9%), and insomnia (18%) of Chinese PHC physicians and nurses reduced by over 13.8% compared with previous studies in the acute phase (i.e., 8th December 2019–11th March 2020), which reported a pooled prevalence of 31.0% for probable depression, 29.0% for probable anxiety, and 13.2%-75.2% for PTSD among physicians (30) and nurses (49). However, the prevalence of probable phobic anxiety (6.2%) and OCD (7.9%) slightly increased compared to previous studies (5.3–5.5%) (8, 50). Increased probable anxiety and OCD could be attributable to the traumatic experiences and fear of infection. A sense of uncontrollability, has been found to be related to increased anxiety symptoms (52), whereas the increased OCD symptoms could be related to personal hygiene and protection measures (6, 51). Our findings further suggested that this sense of uncontrollability might persist or heighten even after the traumatic event. PHC physicians and nurses might practice repetitive personal hygiene measures as compulsions to protect from infection or alleviate distress (6). Moreover, PHC physicians and nurses could be exposed to high levels of suffering and death, leading to heightened phobic anxiety (53). It is important to note that the prevalence of probable somatization (11.2%) was higher than that at the early stage of COVID-19 (5.3%) (8). Our findings suggested that somatization symptoms could be one of the most serious mental health disorders in Chinese PHC physicians and nurses during the post-pandemic period, with stress and distress expressed through physical symptoms probably resulting from prior increased workload and demands, exposure to traumatic events, or personal or family stressors related to the pandemic (54).

### 4.2 Risk factors of probable mental health disorders

#### 4.2.1 Sociodemographics

In line with previous studies (4, 15), the current study similarly found that female gender was a risk factor for different mental health disorders. Adding to the mixed previous evidence on age (8, 15), our study found that older age was associated with higher odds of probable somatization but no other mental health disorders. About 22.0% of the respondents reported chronic diseases and 7.5% reported multimorbidity, which was found to be associated with higher odds of common probable mental health disorders (8, 15, 55). Acknowledging the multicollinearity between multimorbidity and probable mental health disorders (55), the current findings should be replicated with the consideration of this issue. Medical professionals could be more sensitive to somatic symptoms, particularly among people experienced age-related deterioration in physical conditions in conjunction with work stress, burnout, and difficulty in adjustment to overtime work. Existing physical and psychiatric issues could further cause chronic pain and functional impairment (55), and increase physical and psychological burden. The PHC physicians and nurses in Central China reported lower odds of disorders which could be explained by their early exposure to the pandemic outbreak, as they received more support from across the nation (7) and could have more psychosocial resources to buffer against mental ill health (56). Contrarily, physicians/nurses in Southwestern China might be more disadvantaged given the region's developing economy and PHC sectors and the lower socioeconomic status of the physicians/nurses (7).

#### 4.2.2 Work-related characteristics

Job duties and responsibilities were associated with higher odds of multiple probable mental health disorders among PHC physicians and nurses, who shouldered the burden of regular community health care services in conjunction with the implementation of national epidemic prevention and control policies (1, 2). The present findings showed that overtime work was associated with higher odds of common probable mental health disorders. The finding extended previous evidence on the positive association between long working hours and mental illnesses in different occupations (10) to PHC physicians and nurses during prolonged strict COVID-19 quarantine in China. Our results were also consistent with previous evidence (44, 57) on the adverse mental health impact of quarantine experiences among physicians/nurses during COVID-19. People within mandatory and stringent quarantine have reported immediate disruptions to their daily routines of work, exercise, and social activities, and various quarantine-induced stressors concerning health, finance, stigma, and employment could persist even after the pandemic and relate to mental health disorders (57, 58). This study added to previous evidence by assessing quarantine experiences that occurred 1–2 years and showing their prolonged adverse mental health impact in a national sample of PHC physicians and nurses.

#### 4.2.3 Lack of workplace social support in primary health care

Social support has been widely recognized as a protective factor that was associated with lower symptoms of depression, anxiety, PTSD (15, 26), burnout, and quality of life among physicians/nurses. The current study provided additional evidence on the important positive link between social support-seeking behaviors and mental health in the often overlooked primary health care settings. Instead of focusing on the social support PHC professionals received, more attention should be given to facilitating their social support-seeking behaviors, which is an essential coping strategy that helps individuals develop more positive and effective coping strategies and psychological qualities, such as self-efficacy and positive appraisal (26). Such proactive coping with different stressors in PHC could contribute to overall psychological health (59). Despite the potentially important role of social support-seeking behaviors, it was previously found that as low as 12.7% of physicians sought help for their mental health during the COVID-19 outbreak (60), consistent with our findings that 10% of PHC physicians/nurses never sought support. Reluctance to seek help for mental health coupled with a shortage of psychologists (60) should be addressed systematically.

The present study has several limitations. First, causality could not be inferred due to the cross-sectional design. Second, our self-report survey method which could not preclude the possibilities of recall and social desirability biases, although the current instruments were validated and widely used. Third, the traumatic event was not specified in our measurement of PTSD, therefore we could not conclude that the scores reflected respondents' experiences with COVID-19 or related occupational conditions (61). Fourth, apart from the workplace, probable mental health disorders of healthcare professionals could be partially attributable to family issues, such as family distress, family support, and having family members infected (23, 62, 63). Fifth, there could be contextual biases or framing effects which may exist in studies targeting a particular workforce (32). Lower prevalence of mental health disorders was reported in occupation-specific populations with larger sample size compared to those collected in surveys, such as military personnel and police. Last but not least, the current study did not assess other common confounding variables that could influence mental health in COVID-19 such as COVID-19 infection history and perception of COVID-19 information release (64).

### 4.3 Conclusion and implications

Notwithstanding the limitations, the present study has several strengths. First, this national survey recruited one of the largest samples of nationally representative primary health care physicians and nurses using the stratified sampling method, covering both urban and rural areas across seven geographical regions and 30 provincial-level administrative regions in China. To the best of our knowledge, this is one of the first national studies on the mental health of PHC physicians and nurses in China. Second, our study extended the current literature by demonstrating the prolonged mental health impact of stringent infection control rules in the post-pandemic period, supplementing existing body of evidence for the early phase of the outbreak, within and beyond China.

Two years after the pandemic outbreak, under the prolonged strict quarantine measures in China, we observed an overall decreased prevalence of probable mental health disorders and insomnia in Chinese primary health care physicians and nurses, except for the increased probable in somatization, phobic anxiety, and obsessive-compulsive disorder. Primary health care workers might suffer long-term somatic symptoms despite the improvement in overall mental health. Burden and disrupted schedules due to overtime work and quarantine increased the odds of probable disorders. Mental health of primary health care professionals who were older, female, holding higher education degrees, or suffering from multimorbidity were more affected, especially those who did not seek social support. Our results call for attention from an organizational level to provide intervention and rehabilitation programs targeting primary health care physicians and nurses in need, so with the goal of enhancing their long-term physical and mental health and preparedness for future public health crises.

## Data availability statement

The raw data supporting the conclusions of this article will be made available by the authors, upon reasonable request.

## Ethics statement

The studies involving humans were approved by The 7th Hospital of Wuhan (220701). The studies were conducted in accordance with the local legislation and institutional requirements. Electronic informed consent for participation in this study was provided before the data collection.

## Author contributions

CL: Conceptualization, Data curation, Formal analysis, Investigation, Methodology, Software, Visualization, Writing – original draft, Writing – review & editing. YZ: Methodology, Conceptualization, Data curation, Project administration, Resources, Writing – review & editing. YG: Conceptualization, Methodology, Writing – review & editing. ZD: Methodology, Writing – review & editing. XC: Methodology, Writing – review & editing. YL: Methodology, Writing – review & editing. WW: Methodology, Writing – review & editing. TJ: Methodology, Writing – review & editing. QZ: Methodology, Writing – review & editing. LN: Methodology, Writing – review & editing. TT: Writing – review & editing. WH: Formal analysis, Methodology, Supervision, Writing – original draft, Writing – review & editing.
