# Supplementary material for: Mental health of primary health care physicians and nurses following prolonged infection control rules: a national survey in China
Source: Front Public Health. 2024 Aug 23;12:1392845. doi: 10.3389/fpubh.2024.1392845 (PMC11377233; doi:10.3389/fpubh.2024.1392845)
Supplement: Supplementary file 1 [file Data_Sheet_1.pdf]

Supplementary Material

Supplementary Material 1

Bivariate analysis of correlates of probable mental health disorders and insomnia

Variable	Psychological distress		Probable somatization		Probable depression		Probable anxiety		Probable phobia anxiety		Probable obsessive-compulsive disorder	
	N (%)	*p*	*N* (%)	*p*	*N* (%)	*p*	*N* (%)	*p*	*N* (%)	*p*	*N* (%)	*p*
Total	489 (11.52%)		476 (11.21%)		349 (8.22%)		250 (5.89%)		263 (6.19%)		336 (7.91%)	
Gender		0.458		0.237		0.556		0.855		0.219		0.731
Male	88 (10.77%)		82 (10.04%)		63 (7.71%)		47 (5.75%)		43 (5.26%)		59 (7.22%)	
Female	401 (11.69%)		394 (11.49%)		296 (8.63%)		203 (5.92%)		220 (6.42%)		277 (8.08%)	
Age		0.198		<.001		0.101		0.333		0.741		0.007
18-34	180 (10.50%)		156 (9.10%)		119 (6.94%)		86 (5.01%)		105 (6.12%)		119 (6.94%)	
35-49	245 (12.50%)		245 (12.50%)		189 (9.64%)		139 (7.09%)		129 (6.58%)		181 (9.23%)	
50 or above	64 (11.21%)		75 (13.13%)		41 (7.18%)		25 (4.38%)		29 (5.08%)		36 (6.30%)	
Marital status		0.566		0.686		0.293		0.695		0.712		0.916
Married	403 (11.65%)		391 (11.30%)		277 (8.01%)		206 (5.96%)		212 (6.13%)		266 (7.69%)	
Unmarried	86 (10.93%)		85 (10.80%)		72 (9.15%)		44 (5.59%)		51 (6.48%)		70 (8.89%)	
Education level		<.001		<.001		<.001		<.001		0.006		<.001
Below bachelor	133 (8.61%)		132 (8.54%)		96 (6.21%)		66 (4.27%)		75 (4.85%)		88 (5.70%)	
Bachelor or above	356 (13.18%)		344 (12.74%)		253 (9.37%)		184 (6.81%)		188 (6.96%)		248 (9.18%)	
Region		0.002		<.001		0.010		0.005		0.002		0.007
Central	137 (9.37%)		127 (8.69%)		98 (6.70%)		70 (4.79%)		71 (4.86%)		95 (6.50%)	
Southeast	173 (12.12%)		175 (12.26%)		126 (8.83%)		81 (5.68%)		89 (6.24%)		116 (8.13%)	
Southwest	88 (13.62%)		84 (13.00%)		56 (8.67%)		47 (7.28%)		44 (6.81%)		59 (9.13%)	
Northeast	41 (12.54%)		41 (12.54%)		30 (9.17%)		22 (6.73%)		26 (7.95%)		29 (8.87%)	
Northwest	50 (13.02%)		49 (12.76%)		39 (10.16%)		30 (7.81%)		33 (8.59%)		37 (9.64%)	
Residence		0.104		0.133		0.449		0.467		0.284		0.336
Urban	423 (11.87%)		411 (11.53%)		298 (8.36%)		214 (6.00%)		227 (6.37%)		293 (8.22%)	
Rural	66 (9.69%)		65 (9.54%)		51 (7.49%)		36 (5.29%)		36 (5.29%)		43 (6.31%)	
Workplace		0.971		0.407		0.742		0.654		0.426		0.227
Village and township clinics	77 (11.48%)		69 (10.28%)		53 (7.90%)		37 (5.51%)		37 (5.51%)		50 (7.45%)	
Community health service center	412 (11.52%)		407 (11.38%)		296 (8.28%)		213 (5.96%)		226 (6.32%)		286 (8. 00%)	
Occupation		0.344		0.048		0.011		0.048		0.930		0.119

Doctor	254	(11.98%)		258	(12.17%)		197	(9.29%)		140	(6.60%)		132	(6.23%)		182	(8.58%)	
Nurse	235	(11.05%)		218	(10.25%)		152	(7.15%)		110	(5.17%)		131	(6.16%)		154	(7.24%)	
Doctor job position			0.472			0.052			0.010			0.045			0.861			0.172
Not applicable	235	(11.05%)		218	(10.25%)		152	(7.15%)		110	(5.17%)		131	(6.16%)		154	(7.24%)	
Yes, non-front-line	15	(17.24%)		11	(12.64%)		7	(8.05%)		5	(5.75%)		4	(4.60%)		6	(6.90%)	
Yes, front-line	239	(11.76%)		247	(12.15%)		190	(9.35%)		135	(6.64%)		128	(6.30%)		176	(8.66%)	
Technician title			0.003			<.001			<.001			0.043			0.605			<.001
No title and junior	211	(10.07%)		195	(9.31%)		142	(6.78%)		107	(5.11%)		125	(5.97%)		145	(6.92%)	
Intermediate	210	(12.71%)		211	(12.77%)		154	(9.32%)		110	(6.66%)		107	(6.48%)		145	(8.78%)	
Associate senior and senior	68	(13.63%)		70	(14.03%)		53	(10.62%)		33	(6.61%)		31	(6.21%)		46	(9.22%)	
Overtime work			<.001			<.001			<.001			<.001			<.001			<.001
Never and sometimes	257	(8.50%)		238	(7.88%)		172	(5.69%)		111	(3.67%)		137	(4.53%)		167	(5.53%)	
More than half the time and almost everyday	232	(18.95%)		238	(19.44%)		177	(14.46%)		139	(11.36%)		126	(10.29%)		169	(13.81%)	
Childcare responsibility			0.048			0.330			0.033			0.007			0.096			0.052
No	184	(10.37%)		189	(10.65%)		127	(7.16%)		84	(4.74%)		97	(5.47%)		124	(6.99%)	
Yes	305	(12.34%)		287	(11.61%)		222	(8.98%)		166	(6.72%)		166	(6.72%)		212	(8.58%)	
Chronic disease			0.197			0.346			0.396			0.712			0.596			0.599
No	369	(11.18%)		362	(10.97%)		265	(8.03%)		192	(5.82%)		201	(6.09%)		256	(7.76%)	
Yes	120	(12.70%)		114	(12.06%)		84	(8.89%)		58	(6.14%)		62	(6.56%)		80	(8.47%)	
Multimorbidity			<.001			<.001			<.001			<.001			<.001			<.001
No	423	(10.77%)		401	(10.21%)		298	(7.59%)		209	(5.32%)		228	(5.81%)		286	(7.28%)	
Yes	66	(20.69%)		75	(23.51%)		51	(15.99%)		41	(12.85%)		35	(10.97%)		50	(15.67%)	
Psychiatric history			<.001			<.001			<.001			<.001			<.001			<.001
No	444	(10.67%)		435	(10.45%)		307	(7.38%)		214	(5.14%)		233	(5.60%)		299	(7.19%)	
Yes	45	(52.94%)		41	(48.24%)		42	(49.41%)		36	(42.35%)		30	(35.29%)		37	(43.53%)	
Afraid of COVID-19			0.501			0.894			0.154			0.292			0.348			0.037
No	430	(11.40%)		422	(11.19%)		302	(8.01%)		217	(5.75%)		229	(6.07%)		287	(7.61%)	
Yes	59	(12.45%)		54	(11.39%)		47	(9.92%)		33	(6.96%)		34	(7.17%)		49	(10.34%)	
Quarantine experience			<.001			<.001			<.001			<.001			<.001			<.001
No	301	(9.67%)		290	(9.32%)		214	(6.87%)		155	(4.98%)		160	(5.14%)		209	(6.71%)	
Yes	188	(16.59%)		186	(16.42%)		135	(11.92%)		95	(8.38%)		103	(9.09%)		127	(11.21%)	
Emotional support from colleagues			<.001			<.001			<.001			<.001			<.001			<.001
Most colleagues care about you	54	(24.77%)		77	(10.65%)		36	(7.60%)		27	(5.34%)		33	(2.37%)		44	(3.16%)	

Some colleagues care about you a lot	246	(19.32%)		137	(21.56%)		87	(19.72%)		56	(16.06%)		63	(4.63%)		82	(6.02%)	
Care a little when encounter difficulties	124	(9.10%)		215	(16.89%)		183	(14.38%)		132	(10.37%)		126	(9.90%)		172	(13.51%)	
Never care about each other	65	(4.67%)		47	(21.56%)		43	(19.72%)		35	(16.06%)		41	(18.81%)		38	(17.43%)	
Emotional support-seeking behavior			<.001			<.001			<.001			<.001			<.001			<.001
Confide their troubles voluntarily	114	(11.19%)		66	(11.59%)		45	(8.15%)		28	(5.52%)		33	(3.24%)		41	(4.02%)	
Speak up if a friend asks	263	(10.76%)		46	(31.1%)		33	(26.22%)		21	(21.95%)		24	(7.50%)		39	(12.19%)	
Confide only one or two people who are very close	51	(15.94%)		262	(10.72%)		185	(7.57%)		129	(5.28%)		135	(5.52%)		175	(7.16%)	
Never confide to anyone	61	(5.99%)		102	(22.03%)		86	(18.57%)		72	(15.55%)		71	(15.33%)		81	(17.49%)	
Instrumental support-seeking behavior			<.001			<.001			<.001			<.001			<.001			<.001
Frequently ask for help when in trouble	118	(26.11%)		101	(9.75%)		59	(6.83%)		42	(4.64%)		46	(2.92%)		66	(4.19%)	
Sometimes ask for help	171	(15.57%)		116	(23.45%)		77	(19.91%)		51	(16.37%)		57	(5.09%)		78	(6.97%)	
Seldom asks for help	105	(9.38%)		153	(13.93%)		123	(11.20%)		83	(7.56%)		84	(7.65%)		111	(10.11%)	
Never ask anyone for help	95	(6.02%)		106	(23.45%)		90	(19.91%)		74	(16.37%)		76	(16.81%)		81	(17.92%)	

(To be continued)

(Continued)

Variable	Probable hostility		Probable interpersonal sensitivity		Probable paranoid ideation		Probable psychoticism		Probable post-traumatic stress disorder		Probable insomnia	
	N (%)	*p*	*N* (%)	*P*	*N* (%)	*P*	*N* (%)	*p*	*N* (%)	*p*	*N* (%)	*P*
Total	384 (9.04%)		288 (6.78%)		262 (6.17%)		297 (6.99%)		292 (6.88%)		766 (18.04%)	
Gender		0.228		0.251		0.562		0.777		0.158		0.731
Male	65 (7.96%)		48 (5.88%)		54 (6.61%)		59 (7.22%)		47 (5.75%)		144 (17.63%)	
Female	319 (9.30%)		240 (7.00%)		208 (6.07%)		238 (6.94%)		245 (7.14%)		622 (18.14%)	
Age		0.597		0.661		0.617		0.546		0.538		0.007
18-34	139 (8.10%)		108 (6.30%)		94 (5.48%)		107 (6.24%)		104 (6.06%)		272 (15.86%)	
35-49	208 (10.61%)		147 (7.50%)		143 (7.30%)		160 (8.16%)		160 (8.16%)		386 (19.69%)	
50 or above	37 (6.48%)		33 (5.78%)		25 (4.38%)		30 (5.25%)		28 (4.90%)		108 (18.91%)	
Marital status		0.565		0.708		0.689		0.751		0.359		0.916
Married	317 (9.16%)		237 (6.85%)		211 (6.10%)		244 (7.05%)		232 (6.71%)		623 (18.01%)	
Unmarried	67 (8.51%)		51 (6.46%)		51 (6.48%)		53 (6.73%)		60 (7.62%)		143 (18.17%)	
Education level		<0.001		0.017		<0.001		0.008		0.007		<.001
Below bachelor	106 (6.86%)		86 (5.57%)		66 (4.27%)		87 (5.63%)		85 (5.50%)		228 (14.76%)	
Bachelor or above	278 (10.29%)		202 (7.48%)		196 (7.26%)		210 (7.77%)		207 (7.66%)		538 (19.92%)	
Region		<0.001		<0.001		<0.001		<0.001		0.020		0.035
Central	99 (6.77%)		71 (4.86%)		65 (4.45%)		73 (4.99%)		77 (5.27%)		233 (15.94%)	
Southeast	127 (8.90%)		93 (6.52%)		89 (6.24%)		106 (7.43%)		113 (7.92%)		274 (19.20%)	
Southwest	79 (12.23%)		59 (9.13%)		55 (8.51%)		56 (8.67%)		51 (7.89%)		126 (19.50%)	
Northeast	43 (13.15%)		34 (10.40%)		25 (7.65%)		32 (9.79%)		22 (6.73%)		62 (18.96%)	
Northwest	36 (9.38%)		31 (8.07%)		28 (7.29%)		30 (7.81%)		29 (7.55%)		71 (18.49%)	
Residence		0.091		0.388		0.600		0.551		0.891		0.336
Urban	334 (9.37%)		247 (6.93%)		223 (6.26%)		253 (7.10%)		146 (4.10%)		652 (18.29%)	
Rural	50 (7.34%)		41 (6.02%)		39 (5.73%)		44 (6.46%)		46 (6.75%)		114 (16.74%)	
Workplace		0.492		0.935		0.263		0.516		0.491		0.227
Village and township clinics	56 (8.35%)		46 (6.86%)		35 (5.22%)		43 (6.41%)		42 (6.26%)		110 (16.39%)	
Community health service center	328 (9.17%)		242 (6.77%)		227 (6.35%)		254 (7.10%)		250 (6.99%)		656 (18.35%)	
Occupation		0.127		0.213		0.070		0.198		0.139		0.119
Doctor	206 (9.72%)		154 (7.26%)		145 (6.84%)		159 (7.50%)		158 (7.45%)		402 (18.96%)	
Nurse	178 (8.37%)		134 (6.30%)		117 (5.50%)		138 (6.49%)		134 (6.30%)		364 (17.12%)	
Doctor job position		0.749		0.466		0.656		0.735		0.134		0.172

Not applicable	262	(12.32%)		200	(9.41%)		180	(8.47%)		198	(9.31%)		134	(6.30%)		364	(17.12%)	
Yes, non-front-line	14	(16.09%)		10	(11.49%)		9	(10.34%)		12	(13.79%)		7	(8.05%)		12	(13.79%)	
Yes, front-line	108	(5.31%)		78	(3.84%)		73	(3.59%)		87	(4.28%)		151	(7.43%)		390	(19.18%)	
Technician title			0.089			0.072			0.035			0.028			0.067			<0.001
No title and junior	174	(8.31%)		127	(6.06%)		111	(5.30%)		129	(6.16%)		126	(6.01%)		337	(16.09%)	
Intermediate	159	(9.62%)		123	(7.45%)		118	(7.14%)		126	(7.63%)		132	(7.99%)		325	(19.67%)	
Associate senior and senior	51	(10.22%)		38	(7.62%)		33	(6.61%)		42	(8.42%)		34	(6.81%)		104	(20.84%)	
Overtime work			<0.001			<0.001			<0.001			<0.001			<0.001			<0.001
Never and sometimes	205	(6.78%)		142	(4.70%)		127	(4.20%)		154	(5.10%)		146	(4.83%)		420	(13.90%)	
More than half the time and almost everyday	179	(14.62%)		146	(11.93%)		135	(11.03%)		143	(11.68%)		146	(11.93%)		346	(28.27%)	
Childcare responsibility			0.002			0.017			0.024			0.010			0.461			0.052
No	279	(15.73%)		101	(5.69%)		92	(5.19%)		103	(5.81%)		116	(6.54%)		296	(16.69%)	
Yes	105	(4.25%)		187	(7.56%)		170	(6.88%)		194	(7.85%)		176	(7.12%)		470	(19.01%)	
Chronic disease			0.012			0.081			0.052			0.085			0.058			0.599
No	279	(8.45%)		212	(6.42%)		191	(5.79%)		219	(6.63%)		214	(6.48%)		601	(18.21%)	
Yes	105	(11.11%)		76	(8.04%)		71	(7.51%)		78	(8.25%)		78	(8.25%)		165	(17.46%)	
Multimorbidity			<0.001			<0.001			<0.001			<0.001			<0.001			<0.001
No	333	(8.48%)		248	(6.32%)		223	(5.68%)		255	(6.49%)		252	(6.42%)		678	(17.27%)	
Yes	51	(15.99%)		40	(12.54%)		39	(12.23%)		42	(13.17%)		40	(12.54%)		88	(27.59%)	
Psychiatric history			<0.001			<0.001			<0.001			<0.001			<0.001			<0.001
No	348	(8.36%)		251	(6.03%)		230	(5.53%)		261	(6.27%)		259	(6.22%)		713	(17.14%)	
Yes	36	(42.35%)		37	(43.53%)		32	(37.65%)		36	(42.35%)		33	(38.82%)		53	(62.35%)	
Afraid of COVID-19			0.297			0.581			0.244			0.463			0.106			0.037
No	335	(8.88%)		253	(6.71%)		227	(6.02%)		260	(6.89%)		251	(6.65%)		664	(17.60%)	
Yes	49	(10.34%)		35	(7.38%)		35	(7.38%)		37	(7.81%)		41	(8.65%)		102	(21.52%)	
Quarantine experience			<0.001			<0.001			<0.001			<0.001			<0.001			<0.001
No	138	(4.43%)		110	(3.53%)		105	(3.37%)		117	(3.76%)		176	(5.65%)		491	(15.77%)	
Yes	246	(21.71%)		178	(15.71%)		157	(13.86%)		180	(15.89%)		116	(10.24%)		275	(24.27%)	
Emotional support from colleagues			<0.001			<0.001			<0.001			<0.001			<0.001			<0.001
Most colleagues care about you	54	(3.88%)		34	(2.44%)		22	(1.58%)		34	(2.44%)		37	(2.66%)		135	(9.69%)	
Some colleagues care about you a lot	111	(8.15%)		65	(4.77%)		57	(4.19%)		64	(4.70%)		69	(5.07%)		223	(16.37%)	
Care a little when encounter difficulties	178	(13.98%)		148	(11.63%)		147	(11.55%)		159	(12.49%)		147	(11.55%)		342	(26.87%)	
Never care about each other	41	(18.81%)		41	(18.81%)		36	(16.51%)		40	(18.35%)		39	(17.89%)		66	(30.28%)	

Emotional support-seeking behavior			<0.001			<0.001			<0.001			<0.001			<0.001			<0.001
Confide their troubles voluntarily	57	(5.59%)		37	(3.63%)		28	(2.75%)		36	(3.53%)		38	(3.73%)		108	(10.60%)	
Speak up if a friend asks	31	(9.69%)		27	(8.44%)		24	(7.50%)		27	(8.44%)		25	(7.81%)		71	(22.19%)	
Confide only one or two people who are very close	209	(8.55%)		151	(6.18%)		141	(5.77%)		158	(6.46%)		160	(6.55%)		454	(18.58%)	
Never confide to anyone	87	(18.79%)		73	(15.77%)		69	(14.90%)		76	(16.41%)		69	(14.90%)		133	(28.73%)	
Instrumental support-seeking behavior			<0.001			<0.001			<0.001			<0.001			<0.001			<0.001
Frequently ask for help when in trouble	86	(4.95%)		52	(3.30%)		41	(2.60%)		84	(5.33%)		56	(3.55%)		174	(11.03%)	
Sometimes ask for help	86	(7.69%)		65	(5.81%)		49	(4.38%)		96	(8.58%)		58	(5.54%)		187	(16.71%)	
Seldom asks for help	134	(12.20%)		97	(8.83%)		98	(8.93%)		62	(5.65%)		101	(9.65%)		274	(24.95%)	
Never ask anyone for help	86	(19.03%)		74	(16.37%)		74	(16.37%)		55	(12.17%)		77	(17.04%)		131	(28.98%)	
